# Traditional Chinese Medicine Shi-Bi-Man ameliorates psoriasis via inhibiting IL-23/Th17 axis and CXCL16-mediated endothelial activation

**DOI:** 10.1186/s13020-024-00907-z

**Published:** 2024-03-01

**Authors:** Chenyang Zhang, Xinran Cao, Lixin Zhao, Zitong Ni, Haojie Du, Jiao Qu, Jianxia Zhu, Haiyan Sun, Yang Sun, Zijun Ouyang

**Affiliations:** 1 State Key Laboratory of Pharmaceutical Biotechnology, School of Life Sciences, Nanjing University, 163 Xianlin Avenue, Nanjing, 210023 China; 2 Jinling High School, 169 Zhongshan Road, Nanjing, 210008 China; 3 Shenzhen Sipimo Technology Co., Ltd, Shenzhen, China; 4 School of Food and Drug, Institute of Marine Biomedicine, Shenzhen Polytechnic University, 7098 Liuxian Avenue, Shenzhen, 518055 Guangdong China; 5 Jiangsu Key Laboratory of New Drug Research and Clinical Pharmacy, Xuzhou Medical University, 209 Tongshan Road, Xuzhou, 221004 China

**Keywords:** Psoriasis, IL-23, Th17 axis, Single-cell RNA sequencing, Endothelial cell

## Abstract

**Background:**

Psoriasis is a chronic inflammatory genetic disease, mainly manifesting in the skin. Conventional therapies, such as glucocorticosteroids and corticosteroids, have adverse effects that limit drug use. Hence, it is imperative to identify a new therapeutic strategy that exhibits a favorable safety profile. Shi-Bi-Man (SBM) is a safe herbal supplement sourced from various natural plants, including ginseng, angelica sinensis, polygonum multiflorum, and aloe vera.

**Purpose:**

We aimed to find a potential treatment for psoriasis and investigate the underlying mechanism through which SBM alleviates psoriatic-like skin inflammation in mice.

**Methods:**

We investigated the effects of supplementing with SBM through intragastric administration or smear administration in a murine model of imiquimod-induced psoriasis. The changes in body weight and Psoriasis Area and Severity Index (PASI) score were recorded throughout the entire process. Additionally, we used hematoxylin–eosin staining to observe the skin structure and performed single-cell RNA sequencing to explore the underlying mechanism of SBM in influencing the psoriasis-like phenotype. Immunofluorescence was conducted to verify our findings. Furthermore, reverse transcription-quantitative polymerase chain reaction (RT-qPCR) was employed to investigate the impact of Tetrahydroxy stilbene glycoside (TSG) on the expression levels of* IL23* in HaCaT cells.

**Results:**

SBM remarkably alleviated the psoriasis-like phenotype by inhibiting IL-23/Th17 cell axis. Single-cell RNA sequencing analysis revealed a decrease in the expression of *Il17* and *Il23* in keratinocytes and T cells, concomitant with a reduction in the proportion of Th17 cells. Meanwhile, the activation of endothelial cells was inhibited, accompanied by a decrease in the expression of *Cxcl16*. In vitro, the addition of TSG to HaCaT cells resulted in significant suppression of *IL23* expression stimulated by tumor necrosis factor-alpha (TNF-α).

## Highlights

1. SBM ameliorates imiquimod-induced psoriasis-like phenotype in mice.
2. SBM inhibits IL-23/Th17 axis by decreasing Th17 cell proportion and IL-17 expression in keratinocytes and T cells.
3. SBM inhibits CXCL16-mediated endothelial activation.

## Introduction

Psoriasis is a chronic autoimmune skin disease characterized by plaques and scales [1]. The global prevalence is approximately 2% [2]. Despite chronic plaque-type psoriasis, which accounts for about 90% of psoriasis cases, some patients suffer from pustular psoriasis, guttate psoriasis, and inverse psoriasis [3]. Patients with psoriasis have excessive proliferation of keratinocytes, dilation of dermal capillaries, and infiltration of inflammatory cells. Patients afflicted with severe psoriasis experience a range of complications, such as the development of arthritis and immune dysfunction [4]. Dendritic cells and macrophages present in the dermis affected by psoriasis are responsible for the production of interleukin 23. This production leads to the activation of T cells, as well as the release of inflammatory cytokines including IL-17A, IL-17F, IL-22, IL-6, and TNF-α [5]. Small molecule inhibitors and biologics are commonly used in the clinical treatment of psoriasis. In certain case series, the surgical procedure of tonsillectomy has been found to result in a notable amelioration of plaque psoriasis [6–8]. Methotrexate and cyclosporine are widely used in the clinical management of psoriasis. Acitretin is used to inhibit the proliferation and differentiation of keratinocytes [9, 10]. Nevertheless, the attainment of long-term usage is challenging due to the adverse effects and the potential for relapse following cessation [11]. T cells play a pivotal role in mediating various inflammation and immune disorders. T cell-based immunotherapy is advancing. CXC chemokine receptor 6 (CXCR6) has been listed a novel target for immunotherapy for autoimmune, including psoriasis. [12] With the approval of the Etanercept for clinical use by the U.S. Food and Drug Administration, the advent of biologic therapies for psoriasis commenced. Subsequently, the discovery of the IL-23/Th17 axis has led to the development of a growing number of inhibitors that target IL-17 or IL-23, including guselkumab, which have entered the market [13]. Biologic medications, while exhibiting a reduced incidence of adverse effects, impose a significant economic strain on patients due to their exorbitant cost [14]. Hence, it holds clinical importance to identify a treatment that is both safe, efficacious and economically accessible.

As the study of psoriasis has advanced, there has been an increasing recognition of the significant involvement of endothelial cells in the progression of the disease. The characteristic epidermal hyperplasia observed in psoriasis is closely associated with the angiogenic microenvironment influenced by vascular endothelial growth factor (VEGF) [1]. Nonetheless, in another research, psoriasis has been linked to the genetic mutation of VEGF [15]. In addition, suppression of gene expression has been shown to effectively alleviate psoriasis-like features in a mouse model [16]. Furthermore, for psoriatic skin, endothelial cells become activated and release endothelial adhesion molecules, resulting in the recruitment of leukocytes and subsequent initiation of an inflammatory response [17].

As a blend of a variety of Chinese herbs, Shi-Bi-Man (SBM) comprises ingredients such as Radix Ginseng, tea polyphenols, Radix Polygoni Multiflori (the root tuber of *Polygonum multiflorum* Thunb.), Radix Angelica Sinensis from the root of *Angelica sinensis* (Oliv.) Diels, Aloe vera L., linseed, and green tea extract. SBM showed no toxicity in a prior mouse model [18]. Previous research has demonstrated that TSG and EGCG, the primary active components found in SBM, can stimulate hair regrowth by activating the fibroblast growth factor (FGF) pathway in dermal papillary cells [18].

In this study, we aimed to explain the mechanism of SBM in the treatment of psoriasis using single-cell sequencing technology, clarify the target of SBM based on specific cell populations, offering insights that could potentially introduce a new choice for the clinical treatment of psoriasis.

## Materials and methods

### Reagents

Imiquimod (GTH110C, 3 M Health Care Limited, UK) was purchased from Jiangsu Provincial Hospital of Traditional Chinese Medicine. Shi-Bi-Man (SBM) was purchased from Sipimo Biotechnology Co., Ltd (Shenzhen, China). TSG was purchased from Chengdu Purifa Technology Development Co., Ltd. TNF-α was purchased from MedChemExpress USA. Benvitimod was purchased from guanhaobio (Guangdong, China).

### Mice

Six–Eight weeks-old female C57BL/6 mice (2023 g) were purchased from GemPharmatech Co., Ltd (Nanjing, China). The mice were kept in a controlled environment with a 12-h light/dark cycle at a temperature of 25 ± 1 ℃. They were provided with a standard laboratory diet and water ad libitum. The procedures described in this study were approved by the Experimental Animal Care and Use Committee of Nanjing University, by the guidelines outlined in the Guide for the Care and Use of Laboratory Animals (IACUC-2306011).

### Imiquimod-induced model of psoriasis and SBM treatment

After anesthesia with 1% pentobarbital sodium by intraperitoneal injection, the hair on the back of the mice was shaved. A total of 30 mice were divided into six groups: (1) vehicle group; (2) IMQ group; (3) IMQ + benvitimod group; (4) IMQ + SBM group (skin administration; SBM:200 μL, daily for 5 consecutive days); (5) IMQ + 20 mg/kg SBM group (i.g. SBM, daily for 5 consecutive days); (6) IMQ + 40 mg/kg SBM group (i.g. SBM, daily for 5 consecutive days). The vehicle group was treated with glycerol. For the establishment of psoriasis mouse model, mice were topically treated with 62.5 mg 5% IMQ cream on shaved back skin daily for 5 consecutive days. Benvitimod were skin administrated 62.5 mg daily for 5 consecutive days. SBM was dissolved in ddH_2_O containing 5% ethanol.

### Histopathologic assessment

Mouse skin tissue was cut into 5 μm thick pieces. Paraffin sections were successively treated with xylene and ethanol according to the protocol of the hematoxylin–eosin staining kit (G1005, Servicebio, China), and after treatment with hematoxylin solution, eosin staining was used. Neutral balm was used to seal the slides.

### Immunofluorescence

Immunofluorescence staining was performed on tissue and cell samples. For tissue samples, slides were deparaffinized, rehydrated, and treated with sodium citrate buffer for antigen retrieval. Slides were incubated with 3% goat serum for 30 min followed by primary antibodies: CXCL16 antibody (DF13312, Affinity Biosciences, China), VCAM1 antibody (DF6082, Affinity Biosciences, China), SELE/CD62E antibody (DF6914, Affinity Biosciences, China), IL-23A antibody (DF13760, Affinity Biosciences, China), IL-17A Polyclonal antibody (26163-1-AP, PTG, China), Purified anti-human/mouse CD3ε (362701, Biolegend, USA), CD31 antibody (ab134168, Abcam, USA), Anti-E Cadherin antibody (ab231303, Abcam, USA). The secondary antibody used were Goat anti-Rabbit IgG (H + L) Cross-Adsorbed Secondary Antibody, Alexa Fluor™ 488 (A-11008, Invitrogen), Goat anti-Mouse IgG (H + L) Cross-Adsorbed Secondary Antibody, Alexa Fluor™ 594 (A-11005, Invitrogen).

### Realtime-quantitative polymerase chain reaction (RT-qPCR)

TRIzol reagent (Takara, Cat. #9109) was used for total mRNA isolation. We synthesized cDNA using iScript Reverse Transcription Supermix (Bio-Rad), and BioRad CFX96 ouch™ Real-Time PCR Detection System (BioRad, CA, USA) was used for quantitative RT-PCR. HiScript II Q RT SuperMix for qPCR (R223-01) and Taq Pro Universal SYBR qPCR Master Mix (Q712-02) were purchased from Vazyme. Relative gene expression was calculated as 2^^−△△Ct^, where *Gapdh* was used as the housekeeping gene for normalization.

Detailed information about the primers for mice used is listed below:

*Gapdh*, 5′-AGGTCGGTGTGAACGGATTTG-3′ (forward)

5′-GGGGTCGTTGATGGCAACA-3′ (reverse);

*l17f*, 5′-TGCTACTGTTGATGTTGGGAC-3′ (forward)

5′-CAGAAATGCCCTGGTTTTGGT-3′ (reverse);

*Il23*, 5′-ATGAGTTTTTCCCTTATGGGGAC-3′ (forward)

5′-GCTGGAAGTTGGACACCTCAA-3′ (reverse);

Detailed information about the primers for HaCaT cells used is listed below:

*GAPDH*, 5’-GGAGCGAGATCCCTCCAAAAT (forward)

5’-GGCTGTTGTCATACTTCTCATGG (reverse);

*TNF-α*, 5′- CCTCTCTCTAATCAGCCCTCTG-3′ (forward)

5′- GAGGACCTGGGAGTAGATGAG-3′ (reverse);

*IL23*, 5′- CTCAGGGACAACAGTCAGTTC-3′ (forward)

5′- ACAGGGCTATCAGGGAGCA-3′ (reverse);

### Scoring severity of skin inflammation

The clinical PASI was applied to evaluate the progression of psoriasis in mice. According to the PASI scoring standard, erythema, scaling, and thickening were taken into account, each ranging from 0 to 4. 0, none; 1, slight; 2, moderate; 3, significant; 4, extremely significant.

### Single-cell RNA-seq

Skin tissues were collected after euthanasia of the mice and rinsed with phosphate-buffered saline (PBS) thrice. Subsequently, the tissues were sectioned into smaller pieces and digested into single-cell suspensions using collagenase I (Sigma), collagenase II (Sigma), and Dispase^®^ (Sigma).

The suspension was loaded into microfluidic devices using the Singleron Matrix^®^ Single Cell Processing System (Singleron). The scRNA-seq library was constructed according to the protocol of the GEXSCOPE^®^ Single Cell RNA Library Kit (Singleron). The pools were sequenced on a NovaSeq 6000 system (Illumina, USA). The data were uploaded to the GEO database.

To process the raw data and convert it into a matrix suitable for analysis in R, the CeleScope package (v 1.13.0) was employed. The Seurat package (v3.2.3) in R was used for data analysis. Cells were filtered based on gene expression levels, with a threshold of more than 3000 and less than 300 genes. Additionally, cells with mitochondrial reads exceeding 8% and cells with unique molecular identifier (UMI) counts below 800 were excluded. The 'vst' method was used to integrate the data to remove the batch effect. The RunPCA function was used for dimension reduction. We used the uniform manifold approximation and projection (UMAP) function and the FindAllMarkers function. The top 30 genes scored by log2 fold-change were used for cell definition. The R package CellChat (v 1.5.0) was used for cell–cell communication analysis.

### Cell culture and treatment

The HaCaT cell line was procured from the BeNa Culture Collection in Suzhou, China. The cells were maintained in Dulbecco's Modified Eagle Medium (DMEM) supplemented with 10% fetal bovine serum and 1% streptomycin-penicillin solution. The cells were incubated in a humidified environment at 37 °C with 5% CO_2_. To induce cellular response, the HaCaT cells were treated with 50 ng/mL of TNF-α and 5 μM of TSG for a duration of 24 h.

### Spectrum analysis

TSG and ginsenoside Rd were purchased from Chengdu Purifa Technology Development Co., Ltd. and were compared with quality scores greater than 98%.

Chromatographic conditions (qualitative) were as follows. LC-40D liquid chromatograph (SHIMADZHU, Japan); Chromatographic column: Kinetex C18 colume (100 X 4.6 mm, 2.6 μm, Phenomenex, USA).

The injection volume was 5 µL using a mobile phase system with mobile phase flow rate of 0.6 mL/min and column temperature of 40 ℃. The mobile phase were (A) 0.025% formic acid water aqueous solution, (B) methanol–acetonitrile (containing 0.025% formic acid) mixture (50:50, v/v). The gradient elution procedure was as follows: 0–2 min, A: 95%; 2–35 min, A: 95–5%; 35–45 min, A: 5%; 45–46 min, A: 5–95%; 46–50 min, A: 95%.

The mass spectrometry conditions were as follows: Acetonitrile, methanol, and formic acid were chromatographically pure (MERCK company), the water was ultrapure water (obtained by Millipore Milli-Q Synthesis System). Electrospray ion source (ESI) negative ion scanning mode was adopted. The gas temperature was maintained at 550 ℃; The curtain gas was set at 35 psi. CAD gas was 7. The spray voltage was set as 4500 V. Declustering potential was set at 80 V and collision energy at 10 V. TOF Mass range was set at *m/z* 50–1500 Da. All data were collected and processed using an UPLC-Q-TOF-MS/MS (SCIEX Zeno TOF 7600, USA).

### Statistical analysis

GraphPad Prism 8 (GraphPad, San Diego) was used for statistical analysis. One-way ANOVA with Tukey’s multiple comparisons and paired or unpaired Student’s *t*-test were applied. Differences at *P* < 0.05 was considered statistically significant (*P < 0.05, **P < 0.01), and ns represents no significance. All data are presented as the mean ± SEM.

## Result

### SBM relieves psoriasis-like phenotype in the mice model

**Fig. 1:**
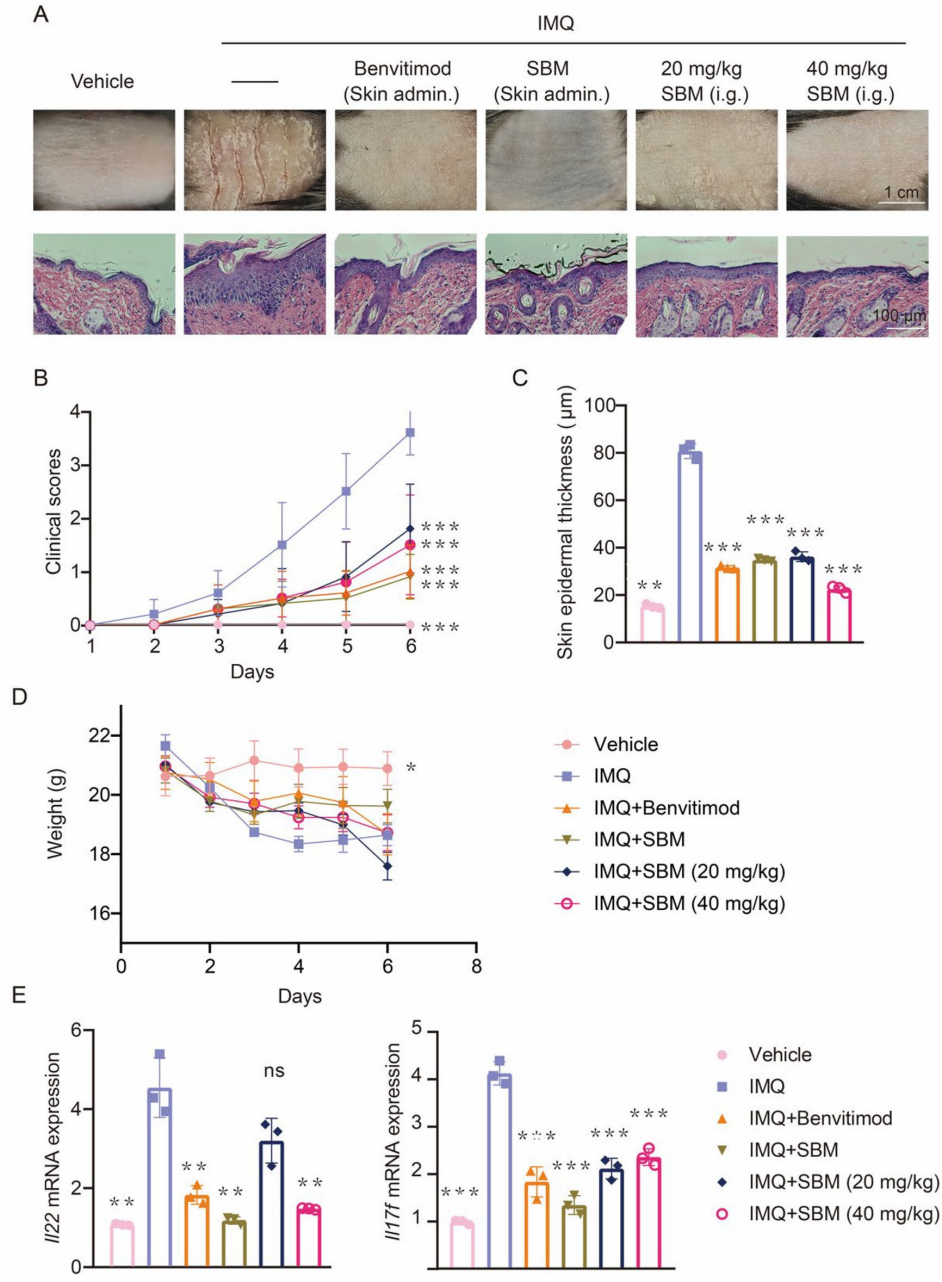
SBM relieves psoriasis-like phenotype in the mice model. **A** The phenotypic presentation and immunohistochemical staining of back skin sections from the Vehicle group, IMQ group, IMQ Benvitimod group (Skin admin.), IMQ + SBM group (Skin admin.), IMQ + 20 mg/kg SBM group (i.g.) and IMQ + 40 mg/kg SBM group (i.g.). **B** The PASI score. **C** The skin epidermal thickness of each group and **D** Weight change. **E** Expression of inflammatory factors IL-17F and IL-22. *P < 0.05, **P < 0.01, ***P < 0.001

To investigate the function of SBM in psoriasis, we applied it to the skin of mice. Benvitimod, which has been shown to have a good therapeutic effect on psoriasis, was used as a control drug [19]. Compared with the IMQ group, SBM alleviated the symptoms of psoriasis induced by IMQ (Fig. 1A). Epidermal thickness was reduced (Fig. 1A–C). The clinical PASI score of the IMQ + SBM group was significantly reduced compared to the IMQ group (Fig. 1B). The body weight (Fig. 1D). In addition, the expressions of inflammatory factors such as IL-17F, and IL-22 were significantly reduced after SBM treatment (Fig. 1E). Among them, the application of SBM solution was the most effective.

### Single-cell RNA sequencing reveals the main skin cell types in mice

**Fig. 2:**
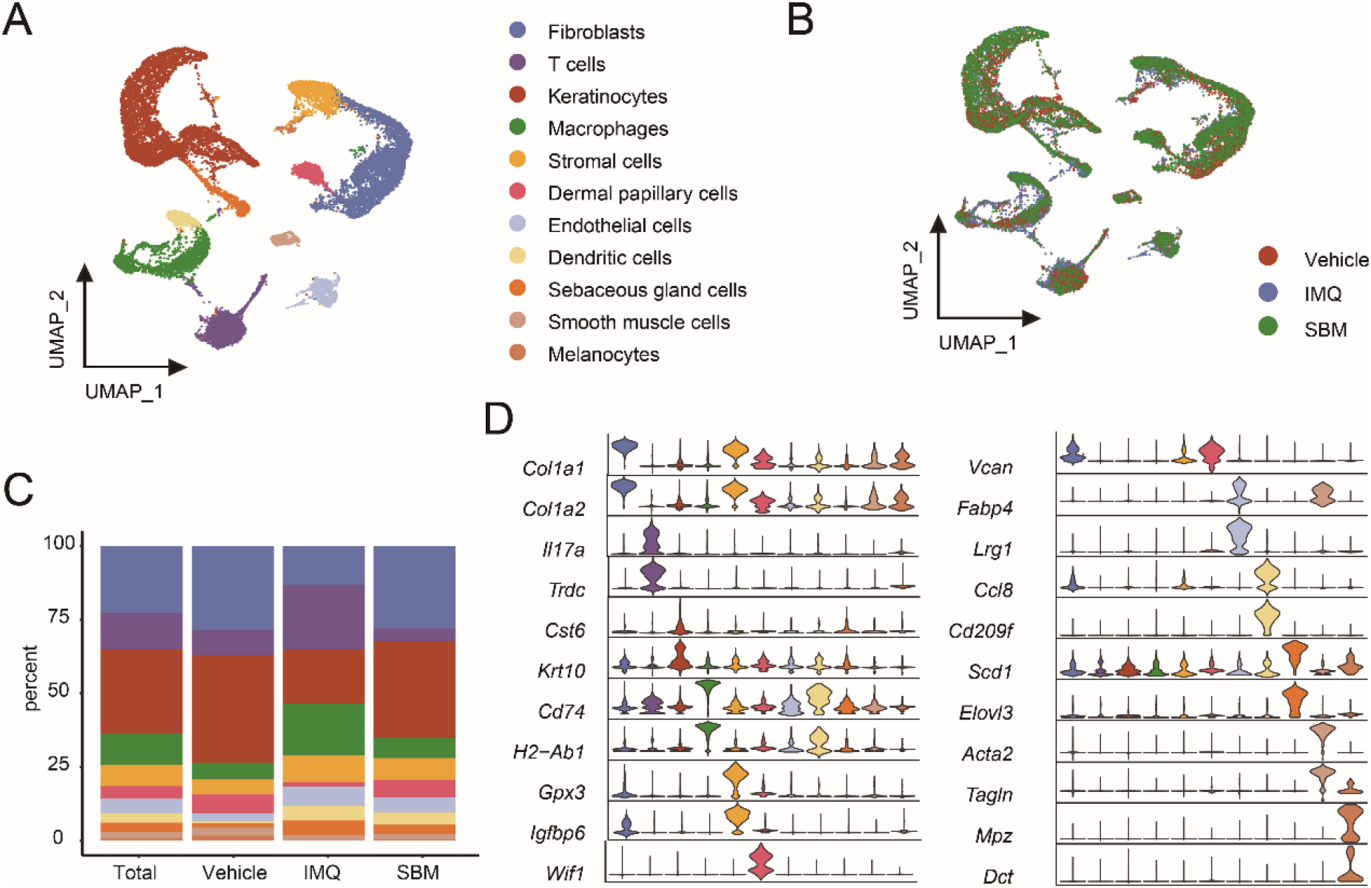
Single-cell RNA sequencing reveals the main skin cell types in mice. **A** The Uniform Manifold Approximation and Projection (UMAP) plot for each cell type. **B** The UMAP for each group. **C** The bar plot for the visualization of the cell proportion of each group. **D** Violin plot for marker genes in each cell type

The single-cell RNA sequencing was performed on the skin tissues of mice. The 11 cell types revealed by unbiased clustering were identified as fibroblasts, keratinocytes, T cells, macrophages, stromal cells, endothelial cells, dermal papilla cells, dendritic cells (DC), melanocytes, smooth muscle cells, and sebaceous gland cells (Fig. 2A, B). The pie chart showed the percent of each cell type (Fig. 2A, B). The cell composition of each group was revealed by a stacked bar diagram (Fig. 2C). The expression of marker genes was presented by a violin diagram (Fig. 2D).

### SBM significantly decreases the expression of psoriasis-related effector genes in keratinocytes

**Fig. 3:**
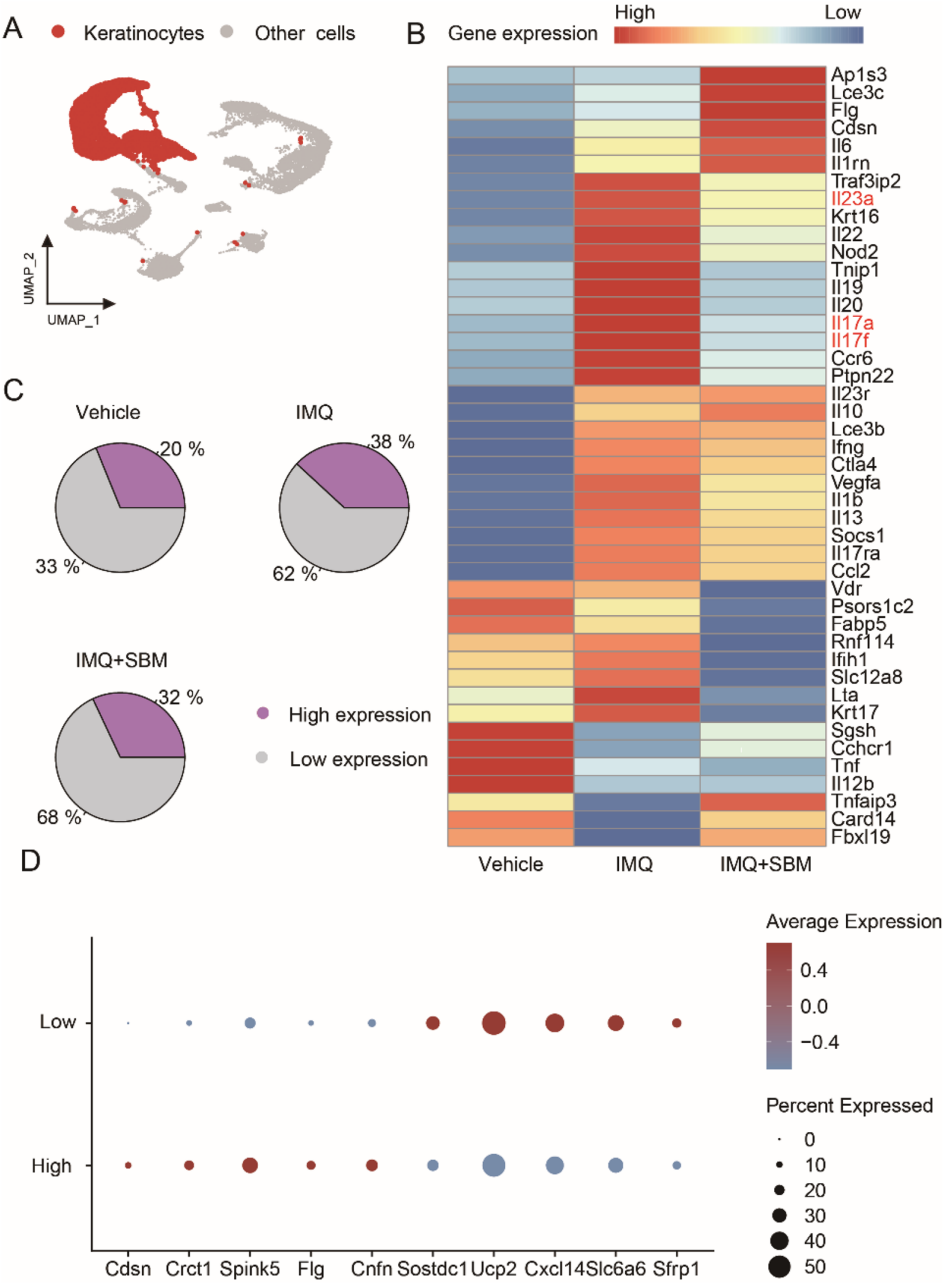
The expression of psoriasis-related genes is downregulated in keratinocytes. **A** UMAP plot for keratinocytes. **B** Heatmap for expression of psoriasis-related genes. **C** Pie chart for percentage of keratinocytes with different psoriasis-related gene expression levels in each group. **D** Dotplot showed the top gene expression in two groups of keratinocytes

Keratinocytes play a crucial role in the pathogenesis and progression of psoriasis. In this study, we isolated and analyzed one keratinocyte subset (Fig. 3A). To identify effector genes which are involved in psoriasis, we used the GeneCards database. The expression of these genes in each group was visualized using a heatmap, with a relevance score threshold of over 20. Interestingly, we observed a downregulation of *Il17a, Il17f* and *Il23a* (Fig. 3B). Based on the expression levels of psoriasis-related genes, we categorized the keratinocytes into two groups: one with high expression and the other devoid of such expression. Following SBM treatment, we observed a reduction in the percentage of keratinocytes exhibiting high expression levels (Fig. 3C). Furthermore, a dotplot analysis revealed the top genes expressed in both keratinocyte groups (Fig. 3D).

### SBM significantly reduces the infiltration of Th17 cells and inhibits the inflammatory response

**Fig. 4:**
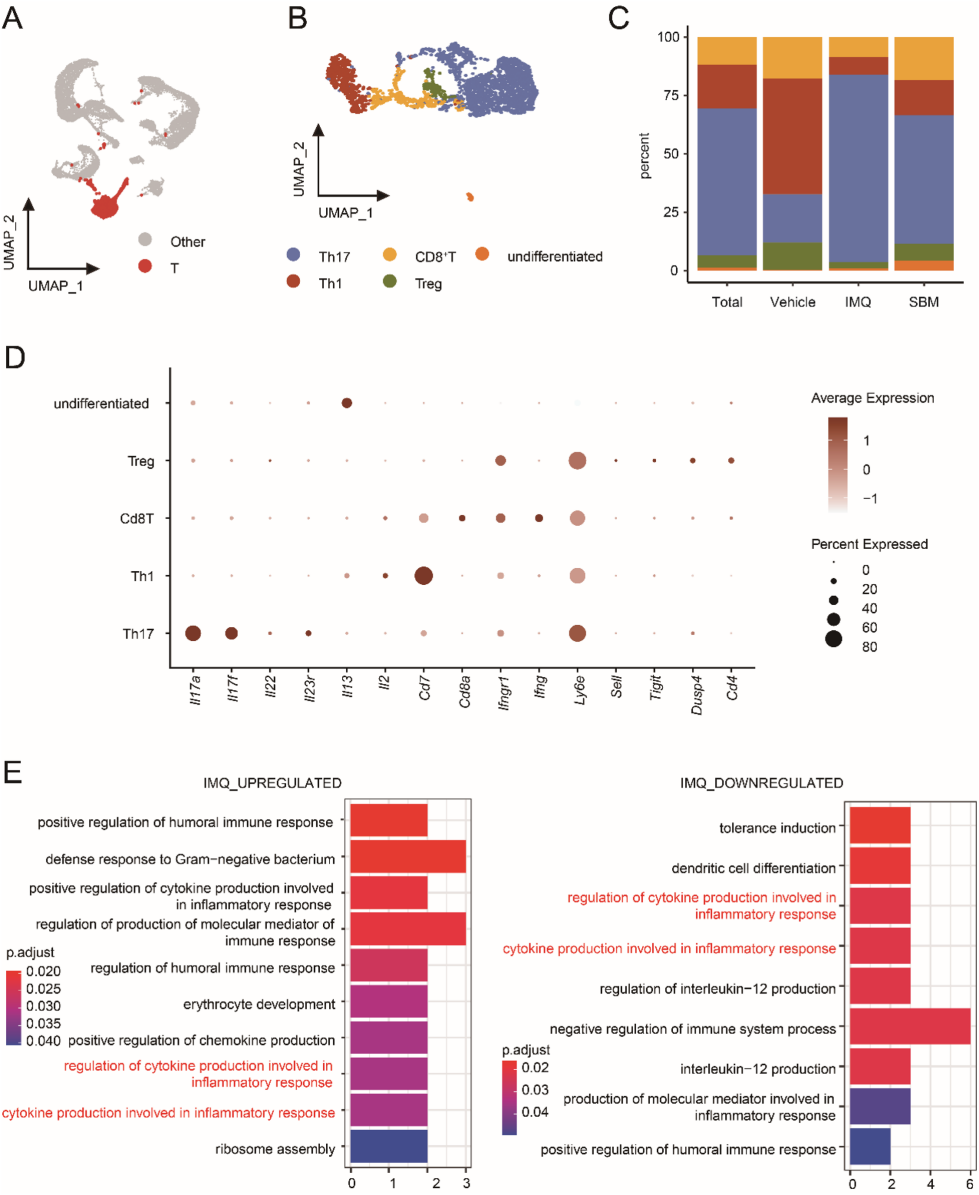
SBM not only suppresses the inflammatory response of T cells but also reduces the proportion of TH17 cells. **A**–**B** UMAP plot for T cells. **C** Stacked barplot for percent of T cells. **D** Dotplot showed the top gene expression in T cells. **E** GO enrichment analysis, including biological process, cellular component, and molecular function

The IL-23/Th17 axis has been proven to be the main mechanism of psoriasis pathogenesis in previous studies. We also found that *Il17a, Il17f,* and *Il23a* were downregulated in keratinocytes after SBM treatment. Thus, we conducted further analysis on T cells and defined the Th17 cells based on the expression levels of *Il17a* and *Il17f* (Fig. 4A–D). The percentage of Th17 cells decreased in the SBM group (Fig. 4C). Gene Ontology (GO) enrichment analysis of the top 100 genes evaluated by the fold change (FC) value (log_2_ FC > 1) with *p*-value < 0.05 showed that SBM treatment suppressed pathways related to inflammatory responses which were upregulated by IMQ (Fig. 4E).

### SBM inhibits the IL-23/Th17 axis

**Fig. 5:**
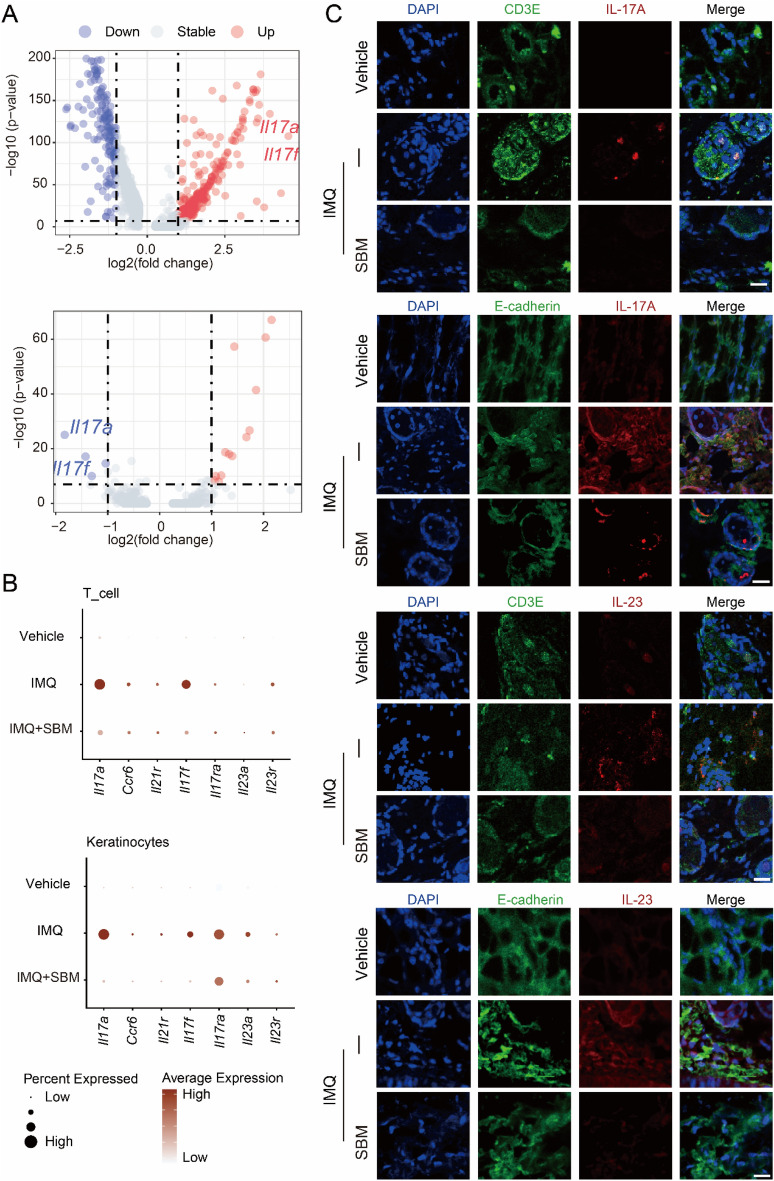
SBM inhibits the IL-23/Th17 axis. **A** Volcano plot for T cells, genes in red represented genes upregulated by IMQ, and genes in blue were downregulated by SBM. **B** Dotplot for the expression of genes related to IL-23/Th17 axis in T cells and keratinocytes. **C** Immunofluorescence images staining for CD3E (green), E-cadherin (green), IL-23 (red), IL-17A (red), and DAPI (blue) of skin tissue, scale bar = 20 μm

Considering the reduction in the percentage of Th17 cells, we suspected that the IL-23/Th17 axis was suppressed. By analyzing the differentially expressed genes, we found that SBM remarkably reversed the upregulation of *Il17a* and *Il17f* (Fig. 5A). The related genes of the IL-23/Th17 axis were downregulated in both keratinocytes and T cells (Fig. 5B). Immunofluorescence confirmed that SBM suppressed IL-23/Th17 axis in psoriasis (Fig. 5C). The infiltration of Th17 was decreased, as shown in Fig. 5C. The keratinocytes expressed less IL-23, which can induce T cell express IL-17. And higher expression level of IL-17 can cause the proliferation of keratinocytes. However, after the expression of *Il17* in T cell was also reduced in Fig. 5A, B. Thus, SBM suppressed IL-23/Th17 axis in psoriasis.

### SBM suppresses endothelial cell activation in psoriasis

**Fig. 6:**
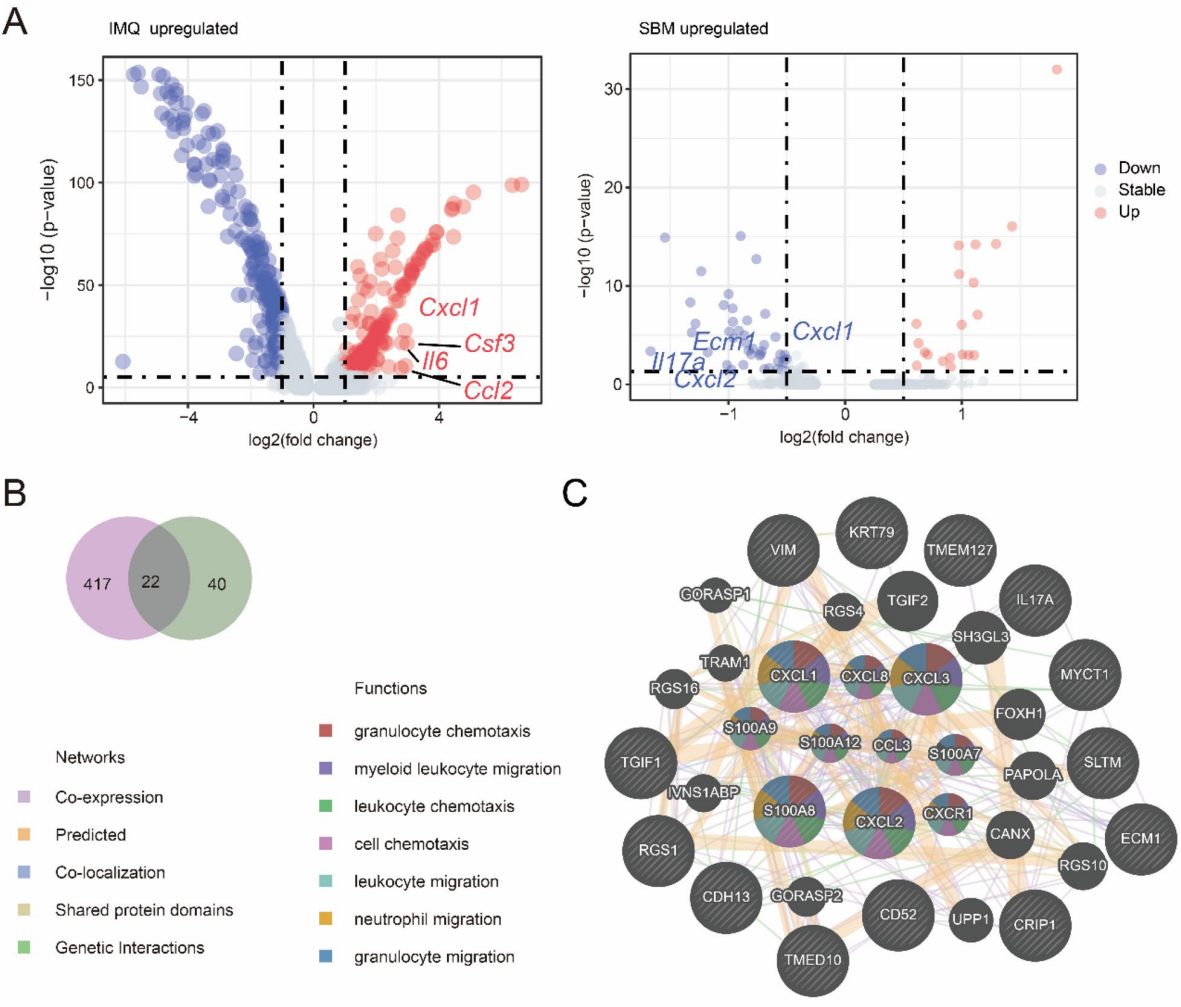
SBM suppresses endothelial cell activation. **A** Volcano plot for endothelial cells, genes in red represented genes upregulated by IMQ, and genes in blue were downregulated by SBM. **B** Venn plots for genes upregulated in the IMQ group and downregulated after SBM treatment. **C** Network diagram of GeneMANIA database analysis

An increasing number of studies have confirmed the importance of endothelial cells in psoriasis [20]. SBM treatment downregulated proinflammatory cytokine genes such as *Cxcl1*, *Il17a* and *Ecm1* (Fig. 6A). We performed GeneMANIA database analysis based on the 22 genes that were upregulated in the IMQ group and downregulated after SBM treatment (Fig. 6B, C). The function of endothelial cells to other immune cells was inhibited, such as myeloid leukocyte migration, cell chemotaxis, leukocyte migration, etc.

### SBM inhibits the expression of Cxcl16 in endothelial cell

**Fig. 7:**
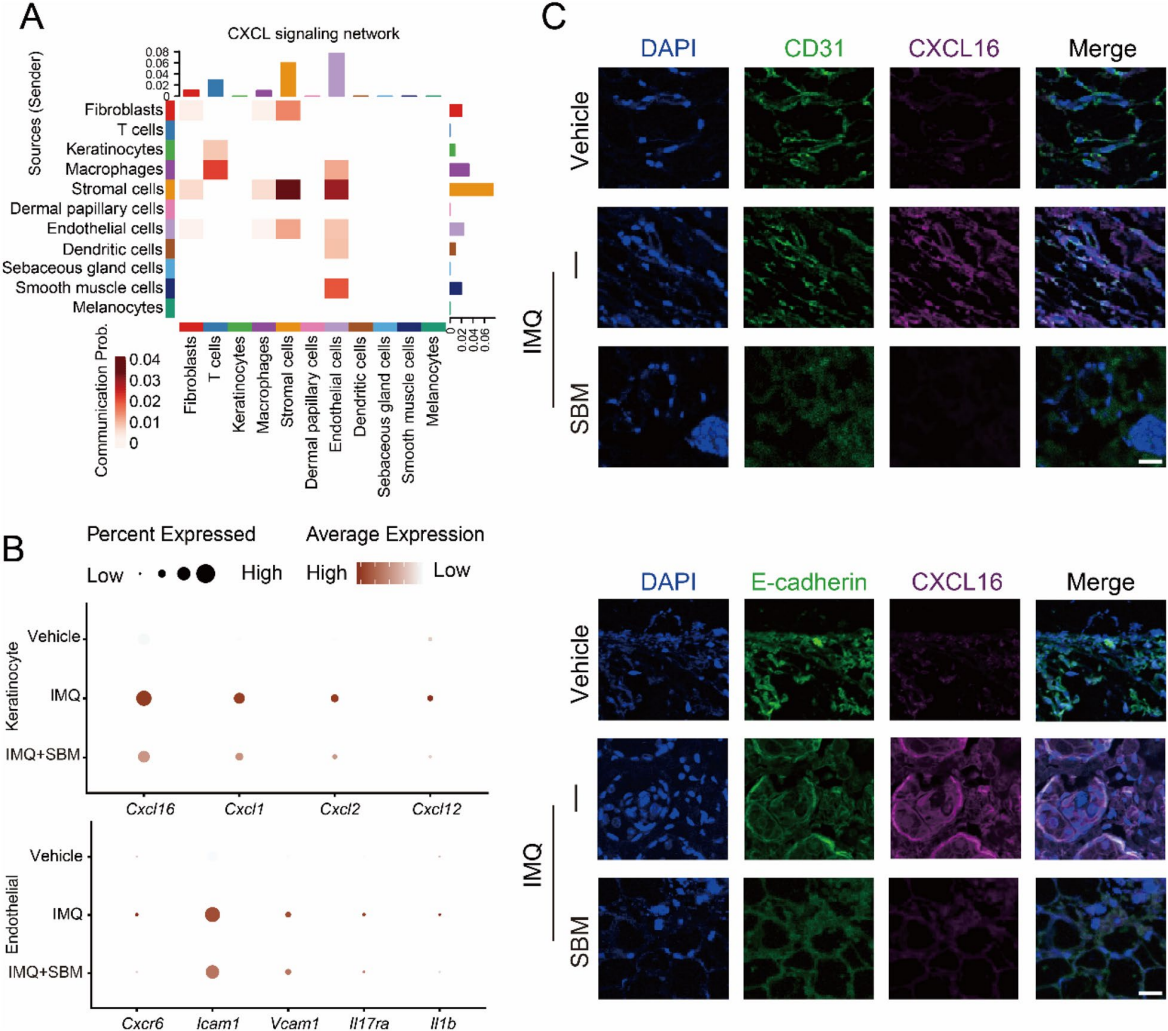
SBM inhibits endothelial cells inflammatory response through *Cxcl16*. **A** Heatmap for CXCL signaling network. **B** Dotplot for the expression of genes related to CXCL signaling in endothelial cells and keratinocytes. **C** Immunofluorescence images staining for CD31 (green), CXCL16 (red), and DAPI (blue) of skin tissue, scale bar = 20 μm

To find out the mechanism of SBM inhibition of endothelial cell activation, we used the R package CellChat to analyze cell–cell communication. Endothelial cells participated in the CXCL signaling network (Fig. 7A). In the IMQ group, keratinocytes expressed higher levels of *Cxcl16*, as well as the expression level of its receptor CXCR6 in endothelial cells. The situation was reversed after SBM treatment (Fig. 7B) and confirmed by immunofluorescence (Fig. 7C).

### TSG can inhibit the expression of Il23 in HaCaT cells

**Fig. 8:**
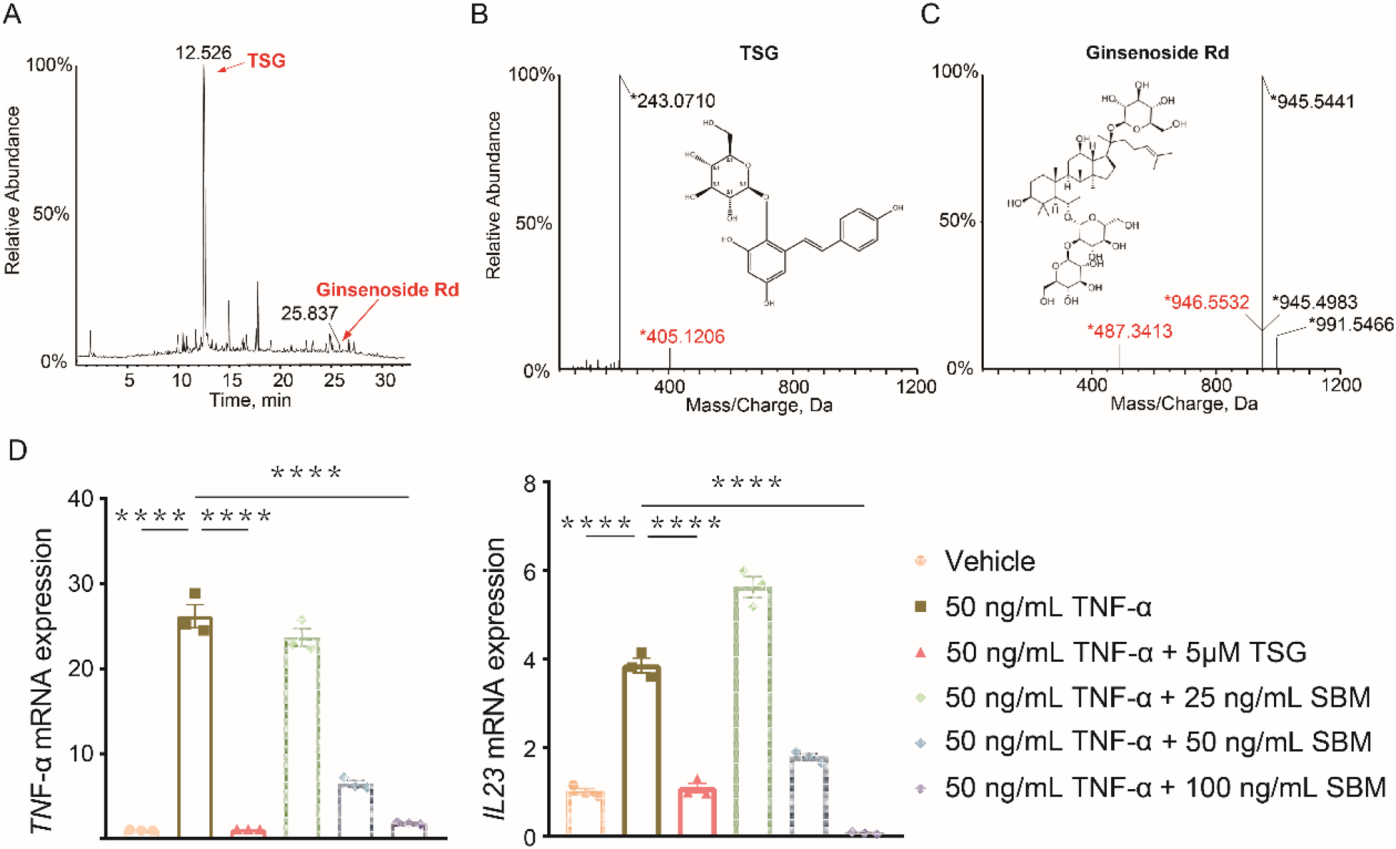
TSG inhibited the expression level of *TNF-α* and *IL23.* TSG and ginsenoside Rd in SBM were detected. **A** Chromatography profile of full scan UPLC/Q-TOF–MS/MS analysis for SBM sample in negative ion mode. **B**–**C** MS spectrum of the identified components in SBM from LC–MS dataset including TSG (**B**) and ginsenoside Rd. **D** Relative mRNA expression level analysis of *TNF-α* and *IL23*. *GAPDH* was used for standardization. HaCaT cells were stimulated with/without 50 ng/mL TNF-α and 5 μM TSG or 25, 50, 100 ng/mL SBM for 24 h. *P < 0.05, **P < 0.01 vs. vehicle group by one-way ANOVA with Tukey's multiple comparisons

As we have identified the main ingredients in the SBM solution previously, we aimed to verify whether TSG and ginsenoside Rd were included in our sample. The UPLC/MS results showed that SBM contained TSG and ginsenoside Rd, and the compound characterization details were shown (Fig. 8A–C). TSG is the major monomer enriched in mice skin after SBM administration [18]. To investigate whether TSG plays a role in psoriasis treatment, we stimulated HaCaT cells with 50 ng/mL TNF-α and 5 μM TSG for 24 h. TNF-α significantly improved the expression of inflammatory factors such as IL-23. However, this was reversed by TSG (Fig. 8D). Since IL-23 promotes infiltration of T cells that produce IL-17 and IL-22 [21], the reduction of *IL23* can inhibit the infiltration of Th17. In addition, the production of IL-17 by Th17 can promote keratinocyte proliferation [22].

**Fig. 9:**
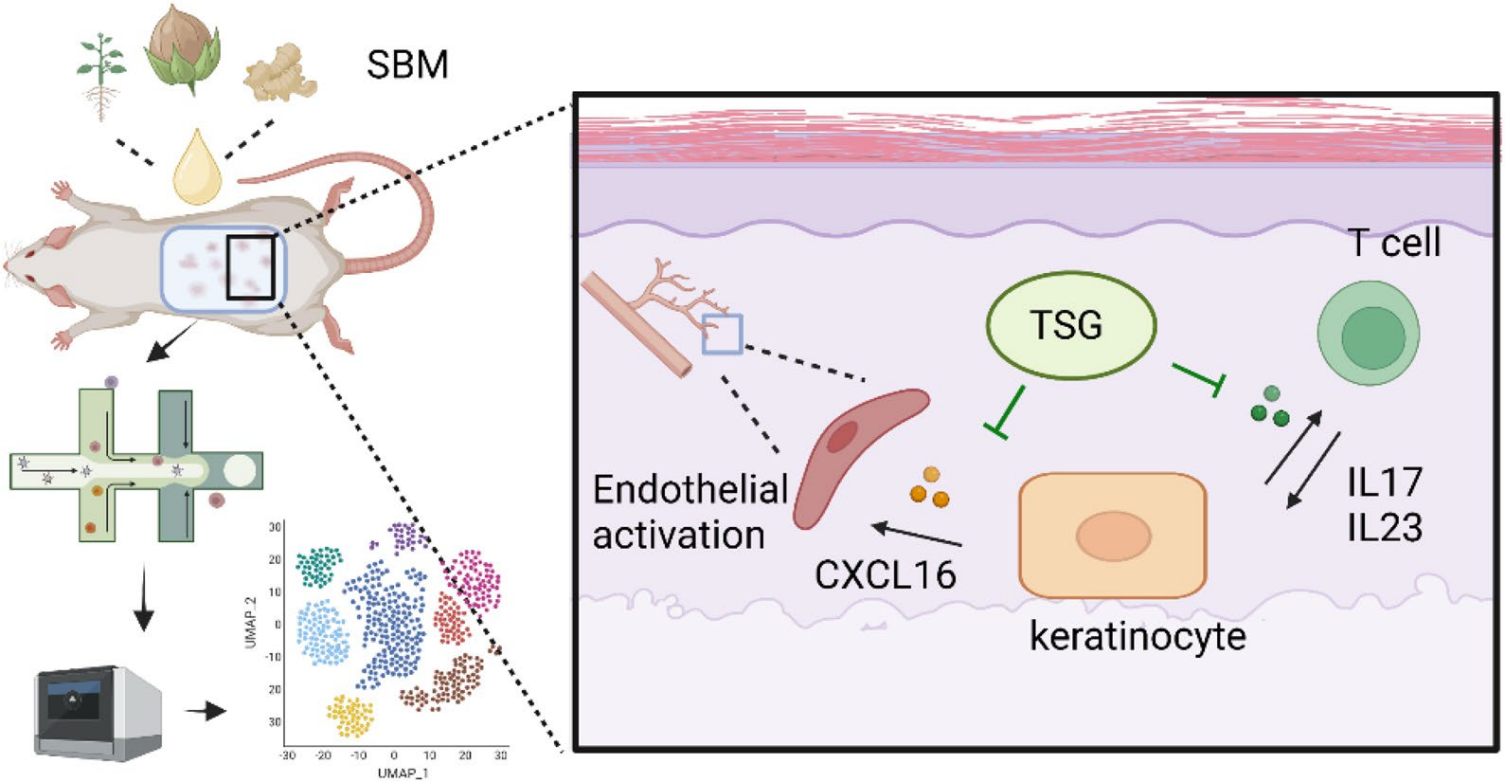
SBM inhibited IL-23/Th17 axis and CXCL16-mediated endothelial activation in psoriasis. Following the implementation of SBM, the expression levels of *Il23* and *Il17* in mouse keratinocytes exhibited a reduction, resulting in a decrease in the proportion of Th17 cells. Additionally, the activation of endothelial cells was effectively suppressed

In conclusion, SBM alleviates the psoriasis-like phenotype by inhibiting the IL-23/Th17 axis and CXCL16-mediated endothelial activation (Fig. 9).

## Discussion

Psoriasis, a chronic relapsing autoimmune skin disease, is influenced by various risk factors. These factors include obesity, dyslipidemia, hypertension, lifestyle choices, and certain medications [23]. Recent researches have established a consensus that the IL-23/Th17 axis plays a crucial role in the development of psoriasis [4, 24]. Despite the emergence of drugs targeting IL-17, further investigation into the underlying mechanisms of psoriasis is ongoing. The severity of psoriasis is associated with the expression of endothelial inflammatory transcripts [25]. Tumor necrosis factor (TNF)-α and other cytokines contribute to the creation of a pro-angiogenic microenvironment in psoriasis [26]. Consequently, VEGF has emerged as a promising therapeutic target for psoriasis [27]. Studies have also demonstrated that alterations in fatty acid metabolism can exacerbate the pathogenesis of psoriasis-like symptoms [28]. Additionally, lysophosphatidic acid, a simple phospholipid found in nature, has been implicated in the development of psoriasis [29]. Furthermore, SHP2 has been shown to exacerbate psoriasis-like skin inflammation in mice through processes such as NETosis or TLR7 activation [30, 31].

As research progresses, it has become evident that a comprehensive understanding of psoriasis pathogenesis necessitates a global perspective that considers the interplay between different cell types and even organs. With the development of research technology, single-cell sequencing technology has gradually entered into basic scientific research [32]. With the development of research technology, single-cell sequencing technology has gradually entered into basic scientific research [33]. Through sequencing technology, we were able to study the molecular mechanism of SBM treatment of psoriasis at the single-cell level and explore the interactions between cells. Based on the analysis results, we can further explore the underlying molecular mechanism of complex diseases and search for potential drugs.

In recent times, there has been a growing utilization of Traditional Chinese Medicine alongside biologics in the management of immune-related disorders [32]. This trend can be attributed to the adverse effects and exorbitant costs associated with biologics, particularly in the treatment of conditions such as psoriasis and other immune diseases. Notably, ginsenoside radix has demonstrated its efficacy in preventing lung injury through its anti-inflammatory and anti-oxidative properties [34]. Re-Du-Ning injection ameliorates radiation-induced pneumonitis and fibrosis [35], as well as lung injury induced by LPS [36]. What’s more, Traditional Chinese Medicine help treat insulin resistance [37]. Paeonol ameliorates endometrial hyperplasia in mice via ferroptosis [38]. The herbal formula also took part in the treatment of psoriasis [39]. Cycloastragenol inhibits NLRP3 inflammasome-mediated pyroptosis in macrophages to relieve imiquimod-induced psoriasis-like skin inflammation in mice [40]. As a mixture of a variety of Chinese herbs, SBM includes ginsenoside radix and has no toxicity using SBM in a mouse model [18]. TSG, the main component in the skin after applying SBM, has been found to have the ability to treat non-alcoholic fatty liver diseases [41]. Furthermore, TSG exhibits anti-aging properties in addition to its anti-inflammatory effects [42]. Therefore, we applied SBM to the IMQ-induced psoriasis-like phenotype mouse model to find a safe and cost-effective treatment for psoriasis patients.

Depending on the scRNA-seq analysis, the potential molecular mechanism of SBM in the treatment of psoriasis was explained. IL-23 induces Th17 cells to activate and release inflammatory cytokines, leading to the typical pathological changes of psoriatic epidermal hyperplasia [43]. The keratinocytes secreted less IL-23 after the treatment of SBM, which leads to less Infiltration of T cells that secrete IL-17 that promotes keratinocytes proliferation [22]. After the treatment of SBM, IL-23/Th17 axis was inhibited. Since IL-17 also promotes endothelial dysfunction, we analyzed the changes in endothelial cells and found the activation of endothelial cells was suppressed. Considering patients with psoriasis are at increased risk of cardiovascular disease, SBM may be beneficial for thrombosis [44]. Further research is needed for specific applications in vascular-related diseases.

The single-cell transcriptomics showed the T cells produce less IL-17 after SBM administration. IL-17 induces keratinocyte proliferation [22]. As shown by single-cell transcriptomics, the expression level of inflammatory factors in endothelial cells was decreased, which reflects the inhibition of endothelial activation. Through cell–cell communication analysis, we found the CXCL signaling network changed (Fig. 7A). In the IMQ group, keratinocytes expressed higher levels of *Cxcl16*, as well as the expression level of its receptor CXCR6 in endothelial cells, which reversed after SBM treatment (Fig. 7B) and confirmed by immunofluorescence (Fig. 7C). We suspected the higher expression of *Cxcl16* in keratinocytes leads to the activation of endothelial cells. And psoriasis is associated with endothelial activation [45].

## Discussion

In conclusion, our results report that SBM significantly alleviated the psoriatic skin inflammation induced by imiquimod in mice via inhibiting IL-23/Th17 axis and CXCL16-mediated endothelial activation. Thus, this work suggests the potential therapeutic value of SBM in patients with psoriasis.

## Data Availability

The single-cell transcriptomic data is uploading to the GEO database. The GSE number is GSE196677.

## Abbreviations

EGCG: Epigallocatechin gallate
FGF: Fibroblast growth factor
GO: Gene ontology
IMQ: Imiquimod cream
PASI: Psoriasis area and severity index
RT-qPCR: Reverse transcription-quantitative polymerase chain reaction
SBM: Shi-Bi-Man
TNF-α: Tumor necrosis factor-alpha
TSG: Tetrahydroxy stilbene glycoside
UMAP: Uniform manifold approximation and projection
VEGF: Vascular endothelial growth factor
